# Soledad, ansiedad y depresión en la adopción del rol de cuidador familiar del paciente crónico*

**DOI:** 10.15649/cuidarte.2451

**Published:** 2023-05-27

**Authors:** Doris Amparo Parada-Rico, Sonia Carreño-Moreno, Olivia Lorena Chaparro-Díaz

**Affiliations:** 1 . Universidad Francisco de Paula Santander, Cúcuta, Colombia. Email: dorisparada@ufps.edu.co Universidad Francisco de Paula Santander Universidad Francisco de Paula Santander Cúcuta Colombia dorisparada@ufps.edu.co; 2 . Doctora en Enfermería. Profesora asociada, Facultad de Enfermería, Universidad Nacional de Colombia. Bogotá, Colombia. Email: spcarrenom@unal .edu.co Universidad Nacional de Colombia Facultad de Enfermería Universidad Nacional de Colombia Bogotá Colombia spcarrenom@unal .edu.co; 3 . Doctora en Enfermería. Profesora asociada, Facultad de Enfermería, Universidad Nacional de Colombia. Bogotá, Colombia. Email: olchaparrod@unal.edu.co Universidad Nacional de Colombia Facultad de Enfermería Universidad Nacional de Colombia Bogotá Colombia olchaparrod@unal.edu.co

**Keywords:** Cuidadores, Interrelación, Ansiedad, Soledad, Depresión, Caregivers, Interrelation, Anxiety, Loneliness, Depression, Cuidadores, Inter-Relagáo, Ansiedade, Solidáo, Depressáo

## Abstract

**Introducción:**

En dirección a la teoría de Meleis, durante la transición como cuidador familiar, subyacen condiciones que facilitan o limitan la adopción del rol, y pueden afectar la integridad de esta persona.

**Objetivo:**

Determinar la relación entre la depresión, ansiedad y soledad con la adopción del rol de cuidadores familiares de personas con enfermedad crónica en Los Patios - Colombia durante el 2021.

**Materiales:**

Investigación cuantitativa analítica transversal que incluyó 120 pacientes y 120 cuidadores. Se aplicaron escalas de Soledad de University of California at Los Angeles; hospitalaria de ansiedad y depresión, y de adopción del Rol del cuidador a través de la plataforma Google Forms®; el análisis se realizó con el software SPSS versión 24, usando estadísticos de frecuencia, tendencia central y dispersión, así como bivariados tipo Pearson.

**Resultados:**

Se halla correlaciones significativas entre la edad del cuidador con la ansiedad (r=,230; p<0.05) y la depresión (r=,297; p<0.05); las horas que requiere a diario para su cuidado con la ansiedad (r=,255; p<0.05) y depresión del cuidador (r=,328; p<0.05).

**Conclusión:**

En un modelo de regresión lineal, se evidencia que la soledad y adopción del rol del cuidador actúan como predictoras de la ansiedad (p<0.05).

## Abstract (en)

**Introduction:**

In line with Meleis'theory, during the transition to a family caregiver, there are underlying conditions that facilitate or limit the adoption of the role and may affect the person's wholeness.

**Objective:**

To determine the relationship between depression, anxiety, loneliness, and the adoption of the role of family caregivers of people with chronic disease in Los Patios (Colombia) during 2021.

**Materials and Methods:**

Quantitative cross-sectional analytical research that included 120 patients and 120 caregivers. The UCLA (University of California, Los Angeles) Loneliness Scale, the Hospital Anxiety and Depression Scale, and the ROL scale were completed by the participants using Google Forms®. The analysis was performed using SPSS version 24; descriptive statistics such as frequencies, measures of central tendency and dispersion, and bivariate correlation coefficients like Pearson's were calculated.

**Results:**

Significant correlations were found between caregivers' age, anxiety (r = .230; p < 0.05), and depression (r = .297; p < 0.05); and between the hours required daily for caregiving and anxiety (r = .255; p < 0.05) and caregiver's depression (r = .328; p < 0.05).

**Conclusions:**

In a linear regression model, it was evident that loneliness and caregiver role adoption are predictors of anxiety (p < 0.05).

## Resumo

**Introdujo:**

Na diregáo da teoría de Meleis, durante a transigáo como cuidador familiar, existem condigóes subjacentes que facilitam ou limitam a adogáo do papel, podendo afetar a integridade dessa pessoa.

**Objetivo:**

determinar a relagáo entre depressáo, ansiedade e solidáo com a adogáo do papel de cuidador familiar de pessoas com doenga crónica em Los Patios - Colombia durante 2021.

**Materiais:**

Pesquisa quantitativa analítica transversal que incluiu 120 pacientes e 120 cuidadores. Escalas de solidáo universitária foram aplicadas da Califórnia em Los Angeles ; ansiedade e depressáo hospitalar, e adogáo do papel do cuidador por meio da plataforma google Formulários®; A análise foi realizada com o software SPSS versáo 24, utilizando estatísticas de frequéncia, tendencia central e dispersáo, bem como bivariadas do tipo Pearson.

**Resultados:**

Sáo encontradas correlagóes significativas entre a idade do cuidador com ansiedade (r=0,230; p<0,05) e depressáo (r=0,297; p<0,05); as horas diárias necessárias para o cuidado com ansiedade do cuidador (r=0,255; p<0,05) e depressáo (r=0,328; p<0,05).

**Conclusao:**

Em um modelo de regressáo linear, fica evidente que a solidáo e a adogáo do papel de cuidador atuam como preditores de ansiedade (p<0,05).

## Introducción

Las Enfermedades Crónicas no Transmisibles (ECNT) continúan siendo un gran desafío para la agenda pública de los países, especialmente de aquellos con ingresos económicos medios y bajos, tal como lo describen Gallardo y Benavides^1^. Lo anterior, no solo debido a la carga de enfermedad e implicaciones que conlleva para el sistema de salud, los pacientes y sus familias este evento, entre ellas la muerte prematura, la pérdida de años de vida productivos y el alto costo de politratamientos^2^, sino además por la subsecuente carga social y del cuidado, junto a los problemas físicos y emocionales que pueden emerger en las personas que adoptan el rol de cuidador, quienes en su mayoría, lo asumen de modo repentino y sin preparación para esta labor, tal como lo afirman diversos autores^3^^-^^5^.

Así las cosas, las ECNT caracterizadas por su larga duración, y alta morbi mortalidad, que ocasionan el fallecimiento de más de 41 millones de personas anualmente en el mundo^6^, plantean de una parte, un reto orientado a disminuir el riesgo de mortalidad en el paciente por esta causa y mejorar su calidad de vida; pero de otro lado, presentan un panorama en torno a las problemáticas y necesidades de los cuidadores de estos pacientes, puesto que transitan en la adopción de un rol, que inicia con falta de preparación, ausencia de habilidades, y con la necesidad de espacios y tiempos propios del cuidador que riñen con sus rutinas cotidianas^7^^-^^10^.

La situación física y psico social del paciente en condición de cronicidad, su grado de dependencia y capacidad funcional, están relacionadas con la necesidad de cuidado y riesgo de mortalidad^5^^,^^11^. Así la adopción del rol y los efectos en la diada, son subsecuentes a la organización, recursos y respuestas al rol de cuidador, acorde a los procesos personales^12^^-^^14^. En consecuencia, las dinámicas dadas en la interacción paciente-cuidador, involucran características individuales, preparación multidimensional del cuidador, soporte social, entre otras, y confluyen en experiencias de cuidado particulares que implican afectación de forma positiva o negativa para la diada, como para su entorno familiar y social^2^^,^^7^.

En este sentido, se identifica que la adopción del rol de cuidador de persona con enfermedad crónica se va dando a partir de la necesidad de cuidado de un familiar o persona cercana; transita en torno a un entramado de interacciones y discurre en el tiempo hacia la adquisición de nuevos conocimientos, habilidades, relacionamientos y estrategias de afrontamiento con el fin de suplir demandas vitales del otro y ajustarse al rol.

Con relación al cuidador, diversos estudios a nivel mundial evidencian que este, es principalmente un familiar del paciente, no remunerado y de sexo femenino^15^^-^^16^, que brinda un cuidado permanente, y según el apoyo social y la duración del rol, puede sufrir deterioro personal en las dimensiones física, emocional, social y estructural. Todo lo cual, puede derivar en sobrecarga subjetiva, ansiedad y depresión durante el ejercicio de este rol^3^^,^^4^^,^^14^^,^^16^^,^^17^^,^^18^.

Algunos problemas identificados en el cuidador primario incluyen trastornos alimentarios, déficit de autocuidado o del disfrute de tiempo libre y sentimientos de culpa por las dificultades para dedicar mayor tiempo a la persona cuidada^19^^-^^21^. Igualmente se ha descrito que en el transcurso del tiempo que puede originar sensación de agotamiento, trastornos del sueño, sentimientos de soledad, estrés crónico y actitudes negativas hacia la persona cuidada^22^^-^^25^. Así las cosas, la satisfacción con el cuidado recibido y la percepción de la necesidad de cuidado del paciente, se relacionan con el deterioro de la calidad de vida de los cuidadores, incluso con la presencia de ECNT en estos. En relación con la soledad de los cuidadores, existen escasos trabajos que valoran este síntoma, no obstante, se reconoce la necesidad de apoyo que tiene este, tanto de la familia como del nivel institucional para continuar esta tarea^26^^,^^27^.

En ese orden de ideas, e identificando que en el departamento Norte de Santander, lugar en donde se realiza la actual investigación, las ECNT han sido la mayor causa de morbilidad y mortalidad en los últimos ocho años en la población mayor de 50 años^28^, y no existen estudios regionales que analicen el impacto negativo de la experiencia en la adopción del rol del cuidador, se plantea determinar la relación entre la depresión, ansiedad y soledad con la adopción del rol del cuidador en cuidadores familiares de personas con enfermedad crónica en el municipio de Los Patios - Colombia en el 2021.

## Materiales y Métodos

Se realizó un estudio analítico de corte transversal conducido durante el primer semestre de 2021 en el municipio de Los Patios Norte de Santander, Colombia. La información obtenida y validada se exportó al software SPSS versión 24 para su análisis. La base de datos fue almacenada en Mendeley Data^29^.

### Participantes

Se llevó a cabo un muestreo por conveniencia, identificando las personas con enfermedades crónicas que asistían a los Centros de atención en salud, adscritos al Hospital Local de los Patios. De esta forma, los cuidadores familiares fueron contactados para realizarles la invitación a participar en la investigación y solicitar el consentimiento informado a través de llamada telefónica. Una vez obtenido el consentimiento verbal, un formulario de Google con un consentimiento escrito y los instrumentos fue enviado a través de correo electrónico o aplicaciones de chat. Previo al autodiligenciamiento del formulario por parte de los cuidadores familiares, se indagó sobre su apropiación de la tecnología y se dio apoyo para el diligenciamiento por parte de las investigadoras para los casos en que fue requerido.

En el estudio fueron incluidos cuidadores familiares de personas con condiciones crónicas tratados de manera ambulatoria que cumplieron los criterios de: ser el cuidador principal, llevar 6 meses o más ejerciendo el rol de cuidador, tener 18 años o más y tener estatus mental intacto. Se excluyeron, aquellos que recibían remuneración o que tenían a cargo más de un sujeto de cuidado. De acuerdo con las características de las personas con ECNT halladas para el estudio, no hubo cuidadores con menos de seis meses en su rol.

El cálculo de tamaño de muestra se hizo con apoyo en el programa G Power con parámetros de entrada de probabilidad de error tipo 1 de 0.05, poder (1-p) del 90%, tamaño del efecto de 0.3 para un total de 112 cuidadores como mínimo. Al respecto, el estudio contó con una muestra total de 120 cuidadores, lo cual es suficiente en cuanto a tamaño.

### Técnicas e instrumentos

Las variables del perfil de los cuidadores y los sujetos de cuidado se colectaron con la Caracterización. Ficha de caracterización de la diada (persona con enfermedad crónica-Cuidador familiar) GCPC-UN-D^30^, que contiene tres secciones que indagan sobre el perfil sociodemográfico, los aspectos relacionados con la dedicación al cuidado y la autovaloración de la carga. Esta encuesta cuenta con validez de contenido para el contexto colombiano, reportando adecuados índices de acuerdo entre los jueces para la importancia, coherencia, claridad y suficiencia de los ítems.

La ansiedad y depresión se midieron con la escala hospitalaria de ansiedad y depresión HADS^31^adaptada al español en 2015^32^. La escala cuenta con 14 ítems, 7 valoran la ansiedad y 7 la depresión en entornos no psiquiátricos o comunitarios. Cuenta con una escala de medición tipo Likert de cero a tres para un total de 21 puntos por cada subescala. La escala reportó rangos de sensibilidad entre 0.74 y 0.84, especificidad de 0.78 a 0.8. Para la validez de constructo se confirmó su estructura de dos factores a través de un análisis factorial exploratorio con rotación Varimax que explicó el 42.5 de la varianza. Reporto un alfa de Cronbach global de 0.83. Para la interpretación de la escala se puntúa con un valor mínimo cero y valor máximo 21 en cada constructo, considera que, a mayor puntaje, mayor ansiedad y depresión, rangos normales entre cero y siete puntos, dudosos entre 8 y 10 puntos y problema clínico de 11 puntos de adelante.

La soledad fue medida con la escala UCLA (University of California at Los Angeles)^33^, es una escala autodiligenciada que consta de 10 preguntas y estructura factorial unidimensional testeada con una rotación varimax que explico el 71.6% de la varianza. La escala cuenta con un alfa de Cronbach de 0.94. Para efectos de su interpretación se considera que, a mayor puntaje, menos presencia de soledad; los rangos de puntaje indican soledad severa cuando hay 19 o menos puntos, soledad moderada entre 20 y 30 puntos y sin soledad entre 31 y 40 puntos.

La adopción del rol del cuidador se midió con la escala ROL^12^, que evalúa 3 dimensiones: respuestas ante el rol, organización del rol y labores del rol. El instrumento cuenta con 22 ítems con escala de medición tipo Likert que va de uno a cinco. Cuenta con una adecuada validez de contenido en la que se reportaron altos índices de acuerdo entre los evaluadores con Kappa de Fleiss de 0.85 para coherencia, 0.67 para claridad y 0.93 para relevancia. En cuanto a la validez facial, se reportó una comprensibilidad alta con Kappa de Fleiss de 0.977. En cuanto a la validez de constructo se informó una estructura de tres factores, además de un alfa de Cronbach superior a 0.8 para la escala total y sus tres factores. La escala reporta niveles de adopción del rol, a saber: Adopción insuficiente del rol (22 a 60 puntos), Adopción básica del rol (61 a 77 puntos) y Adopción satisfactoria del rol (78 a 110 puntos). La estratificación por niveles de adopción del rol se desarrolló mediante la metodología de Dalenius-hodges en un estudio previo^12^.

Se realizo evaluación funcional por medio de la Escala PULSES^13^ evalúa 6 funciones P = estabilidad de la patología o condición física, U = utilización de miembros superiores, L = locomoción o función de los miembros inferiores, S = función sensorial, E = eliminación o control de esfínteres, S = capacidad de socializar. Reporto una consistencia interna con una Alpha Cronbach de 0.74. El método multivariado mostro una alta convergencia entre los ítems del perfil. La escala incluye una interpretación de dificultad con el cuidado que señala baja disfunción de 6 a 8 puntos, mediana disfunción o dependencia de 9 a 11 y alta dependencia del cuidador de 12 en adelante hasta 24, que significa dependencia total.

### Análisis de resultados

El análisis de datos se condujo con el software SPSS versión 24 licenciado por la Universidad Nacional de Colombia. Para las variables cualitativas se analizaron a partir de frecuencias absolutas y relativas; para las cuantitativas, se usaron media, mínimo, máximo, desviación estándar e intervalos de confianza del 95%. Para determinar las correlaciones, previo cumplimiento de requisitos de normalidad, se analizó el coeficiente de correlación de Pearson. Finalmente, se construyó un modelo de regresión lineal en el que fueron ingresadas la soledad y adopción del rol del cuidador como variables independientes y la soledad como variable dependiente. Además, se realizó diagnóstico de colinealidad en donde se halló un estadístico VIF o Factores de Inflación de la varianza de 1.209 lo que evidencia que no existe colinealidad entre las variables independientes, tal como se pudo evidenciar en los análisis realizados.

### Consideraciones éticas

El estudio contó con aval del Comité de ética de la Facultad de Enfermería de la Universidad Nacional de Colombia (AVAL 028-19).

## Resultados

La Tabla 1 presenta las características de los sujetos de cuidados. Se observa una predominancia de personas con enfermedad crónica de género femenino, con una edad media de 77.8 años, 165 meses de trayectoria de la enfermedad (13 años) y con necesidad de 5 horas diarias de cuidado. El régimen de afiliación en salud que se evidencia, en su mayoría fue el subsidiado; más del 40% en condición de multimorbilidad, con predominancia de enfermedades cardiovasculares, la mayoría tienen un único cuidador, siendo los cuidadores principalmente los hijos.

**Tabla 1:** Características de las personas en condición crónica

Variable	Recuento	Porcentaje
Sexo del paciente		
Femenino	86	71.66
Masculino	34	28.33
Régimen de afiliación del paciente		
Contributivo	3	2.50
Subsidiado	117	97.50
N° de enfermedades del paciente		
1	70	58.33
2	35	29.17
3	12	10.00
5	2	1.67
6	1	0.83
Enfermedades del paciente		
Discapacidad	7	5.83
Enfermedades cardiovasculares	99	82.50
Enfermedades mentales	1	0.83
Enfermedades metabólicas	9	7.50
Enfermedades osteomusculares	3	2.50
Enfermedades respiratorias	1	0.83
PULSES		
6 a 8 puntos - baja dependencia	68	56.67
9 a 11 puntos - mediana dependencia	38	31.67
12 a 24 puntos- dependencia total	14	11.67
El paciente se encuentra ubicado en espacio, tiempo y lugar:		
Estatus mental intacto 0 a 2 errores	105	87.50
Variable	Recuento	8.33
Alteración intelectual mínima 3 a 4 errores	10	8.33
Alteración intelectual moderada 5 a 7 errores	5	4.17
Presencia de un único cuidador	85	70.83
Su cuidador principal es:		
Esposo(a)	17	14.17
Hermano(a)	2	1.67
Hijo(a)	79	65.83
Padre/Madre	6	5.00
Otro	16	13.33
Percepción de carga en la relación con su cuidador		
Una carga alta	23	19.17
Una carga baja	60	50.00
Una carga moderada	37	30.83
Edad. Media ± DE	77.8 ± 9.6	77.8 ± 9.6
Meses que lleva con el diagnóstico. Media ± DE	165.7 ± 124.2	165.7 ± 124.2
Número de horas diarias de cuidado. Media ± DE	5± 2.3	5 ± 2.3

La Tabla 2 presenta las características del cuidador familiar. Se observaron cuidadores con edad media de 50 años, casado en un 38%, con niveles de escolaridad entre primaria y bachillerato en el 75%, de ocupación hogar en el 51%, más del 30% con alguna enfermedad y el 84% cuida a la persona desde el momento del diagnóstico.

**Tabla 2:** Características del cuidador familiar

Variable	Recuento	Porcentaje
Sexo del cuidador		
Femenino	98	81.67
Masculino	22	18.33
Estado civil del cuidador		
Casado(a)	46	38.33
Separado(a)	9	7.50
Soltero(a)	29	24.17
Unión libre	31	25.83
Viudo(a)	5	4.17
Grado máximo de escolaridad del cuidador		
Ninguno	9	7.50
Primaria	46	38.33
Secundaria	44	36.67
Técnica o tecnológica	13	10.83
Universitaria	6	5.00
Posgrado	2	1.67
Variable	Recuento	Porcentaje
Ocupación del cuidador		
Empleado(a)	11	9.17
Estudiante	2	1.67
Hogar	61	50.83
Independiente	44	36.67
Pensionado	2	1.67
Estrato socioeconómico del cuidador		
1	40	33.33
2	76	63.33
3	3	2.50
4	1	0.83
Régimen de afiliación del cuidador		
Contributivo	27	22.50
Ninguno	3	2.50
Subsidiado	90	75.00
Presencia de enfermedad en el cuidador	41	34.17
Enfermedad presente en el Cuidador		
Cáncer	1	0.83
Discapacidad	2	1.67
Enfermedades cardiovasculares	20	16.67
Enfermedades metabólicas	4	3.33
Enfermedades osteomusculares	4	3.33
Enfermedades respiratorias	2	1.67
Ninguna	80	66.67
Otras	7	5.83
Tipo de familia		
Extensa	12	10.00
Monoparental	23	19.17
Nuclear	83	69.17
Núcleo sin hijos o hijas	1	0.83
Solo convive con el cuidador.	1	0.83
Cuidado al familiar desde el momento de su diagnóstico	101	84.17
Convivencia con la persona que cuida	85	70.83
Percepción de la calidad de la relación con su familiar		
Buena	74	61.67
Muy buena	38	31.67
Regular	8	6.67
Edad. Media ± DE	50.4 ± 13.2	50.4 ± 13.2
Meses que lleva con la enfermedad. Media ± DE	113.3 ± 109.8	113.3 ± 109.8

La Tabla 3 a continuación presenta los estadísticos descriptivos de las variables de adopción del rol del cuidador total y por dimensiones, ansiedad, depresión y soledad.

**Tabla 3:** Estadísticos descriptivos de las variables del estudio

Variable	Mínimo	Máximo	Media	DE	IC 95%
Respuestas	18	35	28,00	3,47	27.37 - 28.63
Organización	18	40	30,99	5,25	30.04 - 31.94
Labores	22	35	29,60	2,98	29.06 - 30.14
Total ROL	60	104	88,59	8,17	87.11 - 90.07
Ansiedad	1	18	8,68	3,37	8.07 - 9.28
Depresión	0	15	5,51	3,37	4.9 - 6.12
Soledad	15	39	29,91	5,68	28.88 - 30.94

En cuanto a la clasificación de las variables por niveles, la Tabla 4 presenta la descripción de la adopción del rol, soledad, ansiedad y depresión del cuidador familiar. En su mayoría se observa una alta frecuencia de cuidadores con niveles satisfactorios de adopción del rol, niveles normales o dudosos de ansiedad y depresión, además de niveles moderados de soledad.

**Tabla 4:** Clasificación de las variables de estudio por niveles

Niveles	Porcentaje
Rol	
Adopción insuficiente del rol (22 a 60 puntos)	0,08
Adopción básica del rol (61 a 77 puntos)	7,50
Adopción satisfactoria del rol (78 a 110 puntos)	91,70
Ansiedad	
Normal (0 a 7 puntos)	36,70
Dudoso (8 a 10 puntos)	35,00
Problema Clínico (11 o más puntos)	28,30
Depresión	
Normal (0 a 7 puntos)	71,70
Dudoso (8 a 10 puntos)	16,70
Problema Clínico (11 o más puntos)	11,60
Soledad	
Sin soledad (31 a 40 puntos)	56,70
Soledad moderada (20 a 30 puntos)	35,80
Soledad severa (menor que 20)	7,50

### Correlación entre las variables

La Tabla 5 presenta las correlaciones halladas entre las variables de estudio. Se observaron correlaciones positivas y estadísticamente significativas entre la edad del paciente, el número de horas que requiere a diario para su cuidado y la depresión del cuidador. También entre el número de horas de cuidado diario y la edad del cuidador con la ansiedad, la depresión y soledad. Son llamativas también las correlaciones directas entre niveles altos de adopción del rol del cuidador con niveles bajos de ansiedad, depresión y soledad; además de las correlaciones directas entre los niveles altos de ansiedad, depresión y soledad.

**Tabla 5:** Correlaciones entre variables.

Variables	12	3	4	5	6	7	8
1 Edad del paciente	1 0,357**	0,251**	-0,077	-0,273**	0,038	0,212*	-0,011
2 Número de horas de cuidado diario	1	0,176	0,010	-0,143	0,255**	0,328**	-0,063
3 Edad del cuidador		1	-0,104	-0,316**	0,230*	0,297**	-0,203*
4 Meses que lleva el cuidador enfermo			1	-0,216	0,332*	0,157	-0,189
5 Adopción del rol				1	-0,422**	-0,486**	0,416**
6 Ansiedad					1	0,604**	-0,646**
7 Depresión						1	-0,510**
8 Soledad							1

(*) p <0.05, (**) p <0.001

### Modelos de regresión

La Tabla 6 muestra tres modelos de regresión de las variables de estudio. En el primer modelo se reporta una varianza explicada del 56% con la adopción del rol del cuidador y la soledad como predictores de la ansiedad. El segundo modelo con una varianza explicada del 48% identifica la ansiedad como predictora de la adopción del rol del cuidador y el tercer modelo, con una varianza explicada del 45% en la que la ansiedad se evidencia como predictora de la soledad.

Se construyó un modelo de regresión lineal en donde las variables independientes fueron la adopción del rol del cuidador y la soledad, y, la variable dependiente fue la ansiedad. El modelo tuvo como estadísticos una varianza explicada del 56% es decir un R2=0.56, F=6.3 y p<0.01.

**Tabla 6:** Modelos de regresión Variable dependiente Ansiedad

Variables independientes	B Beta Crudo (IC 95%:19,78-50,18)	ET (Valor p)	p Beta ajustado (IC 95%)	t (Valor p)
Adopción del rol	-0,17 (-0.298/-0.052)	0,06	-0,40(-0.598/-0.122)	(0.007)
Soledad	-0,22 (-0.37/-0.074)	0,07	-0,40(-0.57/-0.144 )	(0.004)

(*) p <0.01

## Discusión

Respecto a las características de los pacientes participantes del estudio, en su mayoría son mujeres con más de 60 años de edad (78%), datos semejantes a los hallazgos de Aguilera, et al.^5^ y Blanco, et al.^3^; un grupo importante presenta multimorbilidades S(42%), no obstante, las enfermedades cardiovasculares predominan como principal afectación, las que a su vez son la principal causa de mortalidad a nivel departamental y nacional^28^^,^^34^. En relación al cuidado necesario, requieren más de una jornada de medio tiempo, y ostentan moderada dependencia de su cuidador.

Lo anterior agrupa variables sociodemográficas y de salud concordantes con resultados de autores a nivel nacional e internacional^17^^,^^35^^,^^36^, que demuestran de una parte, la carga de enfermedad en perspectiva de género, así como la marcada necesidad de apoyo que requieren estos pacientes y su grado de dependencia, lo cual puede tener implicancias en la relación de la diada, en la economía familiar y en la salud del cuidador, puesto que variables como la edad, pluripatología del paciente y grado de dependencia, se relacionan con la adopción del rol y con la presencia de síntomas negativos en la salud del cuidador^1^^,^^22^^,^^27^^,^^37^.

En el estudio se halla que, a mayor edad del paciente y del cuidador, hay menor adopción del rol del cuidador. En efecto, la adopción del rol del cuidador, aunque relacionada, no es un efecto de la edad, lo que puede explicar esto es que a mayor edad puede haber mayor deterioro físico y por tanto más dependencia del paciente y del cuidador, lo que puede dificultar aspectos como la organización del rol y la ejecución de las labores del rol en términos del cuidado directo. Esto, finalmente, dificulta la adopción del rol, en particular las labores y organización, lo que condiciona respuestas negativas y algunas de sus expresiones pueden ser la sobrecarga y la afectación de la calidad de vida.

De otra, se va consolidando una dependencia de cuidado que asume el cuidador, casi en su mayoría desde el momento del diagnóstico (84%). Este cuidador se caracteriza por ser de sexo femenino, coincidiendo con el rol asignado a la mujer desde una lógica cultural, posee una edad media de 50 años, nivel de escolaridad entre primaria y bachillerato, de ocupación hogar, resultados concordantes con estudios a nivel de Latinoamérica y España^4^^,^^17^^,^^19^^,^^36^, pero divergentes de resultados en países como China, como lo evidencian Huang, et al.^25^, en el que los cuidadores tienen un nivel de estudios más alto, comparten el cuidado en su mayoría con otras personas y realizan labores remuneradas en otros espacios y tiempos. También se identifica en los cuidadores del actual estudio que más del 30% de estos, poseen alguna enfermedad, la que aparece alrededor de unos cuatro años posteriores al inicio del cuidado, tal vez en relación con esta labor, lo que podría analizarse en un estudio posterior.

Los resultados permiten valorar la connotación económica y social que enmarca el contexto de los cuidadores familiares en la región de estudio; estos entretejen el cuidado del paciente y labores del hogar, principalmente sin recibir remuneración económica, pero además sus niveles de escolaridad bajos no permiten ostentar un empleo bien remunerado, u obtener recursos para emplear un cuidador que realice esta función. En este sentido, se va demarcando un perfil del cuidador con necesidades económicas y escasos capitales para organizar y dar respuesta al rol demandante de cuidado permanente, al igual que en otros contextos latinos^38^. En este aspecto, en el país se han diseñado normativas orientadas a proporcionar apoyo a los cuidadores, como por ejemplo el proyecto Ley 009 de 2020 del Senado, que reconoce la figura jurídica del cuidador, con beneficios en capacitación, flexibilidad laboral, posibilidad de vincularse al sistema de salud y pensión y un ingreso mínimo vital; se espera que, como resultado de la política con el sistema de cuidado, pueda ser aprobada esta iniciativa de Ley.

Respecto a los hallazgos relacionados con la adopción del rol, se valora la transición del cuidador en las dimensiones organización, labores y respuestas ante el rol, de modo satisfactorio por la mayoría de los participantes (91,7%), de tal forma que existe una adaptación a esta labor en el transcurso del tiempo. También se halló que existe una relación entre los altos niveles de adopción del rol del cuidador con bajos grados de ansiedad, depresión y soledad. Lo que coincide con estudios que miden la utilización de diversas estrategias referentes a la dimensión organización y labores en el rol, entre las que se destacan el diseño de planes de acción a partir de la identificación de las necesidades de cuidado del paciente^17^; la intervención de grupos y programas de apoyo para el cuidado^39^; el sentido de coherencia que involucra la utilización de los recursos^14^, todo lo cual, aporta para la adquisición de habilidades, mejora la experiencia y en consecuencia, la adopción de su rol.

En el estudio se identifican tres modelos de regresión en relación con la adopción del rol del cuidador y la aparición de soledad, ansiedad y depresión. En el primer modelo se reporta una varianza explicada del 56% con la adopción del rol del cuidador y la soledad como predictores de la ansiedad. Estos resultados indican que en la medida en que se asume el rol y se transita hacia su ejecución de una manera adecuada, es decir desarrollando capacidades a partir de las experiencias significativas, y recibiendo el apoyo de otros para continuar trayectorias vitales en medio de las labores del cuidado, el nivel de ansiedad disminuye y se asocia con una mejor calidad de vida y salud mental^20^.

El segundo modelo con una varianza explicada del 48% y la ansiedad como predictora de la adopción del rol del cuidador y el tercer modelo, con una varianza explicada del 45% y la ansiedad como predictora de la soledad. Así según lo referido por Sandín, Chorot, & McNally^40^, los cuidadores con problemas de ansiedad tienen menos interacción con recursos de apoyo y utilizan estrategias de autoculpa, critica y censura, lo cual aumenta sensaciones de soledad. Estos resultados se asocian con los hallazgos de Gavilán^24^, quien resalta que la mayoría de los cuidadores informales en España no tienen apoyo familiar ni institucional suficiente, lo que les supone una fuente de estrés y de cambios en su rol social, aunado a una gran sensación de soledad.

Lo descrito en los resultados del actual estudio, permite identificar la necesidad de privilegiar intervenciones de enfermería con cuidadores familiares de personas con ENT, que conlleven a favorecer la adopción del rol y a mejorar las redes de apoyo para transitar de modo adecuado hacia esta labor como cuidador principal; todo lo cual aporta a disminuir sobrecarga, niveles de ansiedad y sensación de soledad.

## Conclusiones

Se puede concluir que existe una relación entre las variables de impacto negativo en la experiencia de ser cuidador como la ansiedad, depresión y soledad con la adopción del rol del cuidador, todo lo cual implica la forma en que se organizan cotidianamente, los recursos existentes y las respuestas que tienen de su experiencia. Además, se logra identificar que variables sociodemográficas como la edad del paciente y el número de horas del cuidado son elementos clave para la predicción de la depresión y que la adopción del rol del cuidador puede ser una variable de resultado, que afecta el nivel de ansiedad, depresión y percepción de soledad. Esto significa que, si se clarifica, se ensaya y se modela el rol, podría incidir en la disminución del impacto negativo en la labor de cuidado.

Con relación a las limitaciones del estudio, se relacionaron con la muestra intencional obtenida, que no permite generalizar estos datos, sino solo determinar un perfil de los cuidadores en el municipio de Los Patios, Norte de Santander, Colombia. No obstante, esto es útil para políticas locales y para aportar en el marco de la formación de profesionales de enfermería.
